# Distribution characteristics of top SOC in different forest types of Genhe in the Greater Khingan Range of Inner Mongolia

**DOI:** 10.1038/s41598-025-88170-6

**Published:** 2025-02-25

**Authors:** Jie Zhang, Mingyue Song, Shulin Zheng, Pei Zhou, Jun Guo, Junnan He, Haijun Yue

**Affiliations:** 1 College of Energy and Transportation Engineering, Inner Mongolia Agricultural University, Hohhot, 010018 China; 2 School of Transportation and Logistics Engineering, Wuhan University of Technology, Wuhan, 430070 China; 3 College of Forestry, Inner Mongolia Agricultural University, Hohhot, 010018 China

**Keywords:** Top soil, Semivariogram model, Spatial distribution pattern, Different forest types, Forestry, Forest ecology

## Abstract

Soil serves as the largest active carbon reservoir in terrestrial ecosystems, playing a pivotal role in carbon sequestration. The study area is situated in Genhe City, on the western slope of the northern segment of the Greater Khingan Range in Inner Mongolia. We utilized a TOC/TN analyzer to measure the soil organic carbon (SOC) content and applied data analysis methods, including the coefficient of variation and the semivariogram function model, to examine the spatial distribution characteristics of SOC content in the surface layer. The results showed that the variation range of SOC content in the 0–5 cm and 5–10 cm soil layers of the four types of forest land sampling points is 100.98–327.59 g/kg and 44.22–322.71 g/kg. Within the same forest land, SOC content decreases with increasing soil depth. Overall, as latitude increases, the top SOC content shows an upward trend. However, in the Hanma region, the relationship between SOC content and latitude varies across different soil layers. This variation may be attributed to differences in vegetation, topography, and altitude. We believe that latitude, forest type, and altitude all influence the spatial distribution of SOC content. These findings provide a scientific basis and reference for establishing the spatial distribution patterns of top SOC content.

## Introduction

Forest soil organic carbon (SOC) is the most critical component of the forest soil carbon pool. Its content regulates forest soil fertility, provides energy and nutrients for the growth of trees and soil microorganisms^1^. As the largest carbon pool in terrestrial ecosystems, the organic carbon content in soil is at least three times that in terrestrial plants or the atmosphere^2^. Given its crucial role in restoring soil health and atmospheric carbon sequestration^3^, experts and scholars increasingly focus on soil’s significant CO_2_ absorption and retention capacity^4^.

The SOC pool exhibits high spatial heterogeneity, significantly impacting soil structure, function, and vegetation spatial distribution at different scales^5^. Research on soil spatial heterogeneity involves various land-use types^6^, different regions^7^, and diverse forest stand types^8,9^. Yuan et al.^10^ analyzed the soil physical and chemical characteristics of mixed and pure forests, concluding that forest stand type is one of the key factors affecting the soil properties of Zijin Mountain.

Some studies also indicate that topographical factors, such as latitude, altitude, temperature, and soil depth, influence SOC content^11,12^. Wu et al.^13^ when exploring the vertical distribution pattern of SOC in natural primary forests and secondary forests in Hainan, note that the SOC content in the study area increases with altitude. Wang et al.^14^ point out that understory SOC shows significant top aggregation and decreases exponentially with increasing soil depth in their study on SOC characteristics of larch forests. Additionally, SOC content is significantly positively correlated with soil moisture content, ammonium nitrogen, available potassium, and organic phosphorus content. Therefore, the spatial distribution of SOC content varies among different forest types, leading to research outcomes with distinct regional characteristics and irreplaceability. Consequently, studying the spatial distribution of SOC in different regions is necessary, providing theoretical references for enhancing the carbon sequestration capacity and nutrient availability of forest soils.

Applying geostatistics in SOC research can explore its spatial variation characteristics and influencing factors^15,16^. Mallik et al.^17^ use remote sensing data and topographical data, combined with geostatistics, for mapping and predicting SOC. Du et al.^18^ employ geostatistical methods to describe the spatial distribution of SOC density in Guangxi forests, which indicates that the best-fit model is the exponential model, showing moderate spatial autocorrelation. This suggests that SOC density in the study area is mainly influenced by structural factors such as soil parent material, topography, and climate rather than by human activities. Yu^19^ comparative analysis of the spatial variation characteristics of SOC in different permafrost regions of the Huma Basin using geostatistical methods.

The Greater Khingan Range forest area, located in the northeastern part of the Inner Mongolia Autonomous Region, is one of China’s four major state-owned forest areas, covering a total area of 106,700 square kilometers. The forest coverage rate in this area reaches 79.56%, ranking first in terms of total forest area and stock volume among all state-owned forest areas in china^20^. Therefore, this paper hypothesizes that the spatial heterogeneity of SOC in different forest land types in the Genhe forest region of the Greater Xing’an Mountains in Inner Mongolia is closely related to their ecological characteristics. Furthermore, significant differences in SOC distribution are expected across different soil layers. This study applies geostatistical methods to analyze the spatial distribution characteristics of SOC distribution are explores the influence of different forest land types on SOC content. The research provides new data support for the assessment of top soil carbon stocks in the region, particularly in terms of the spatial distribution of carbon storage potential, and offers an important data foundation for the evaluation and management of forest soil carbon stocks in the region.

## Materials and methods

### Study area

**Fig. 1:**
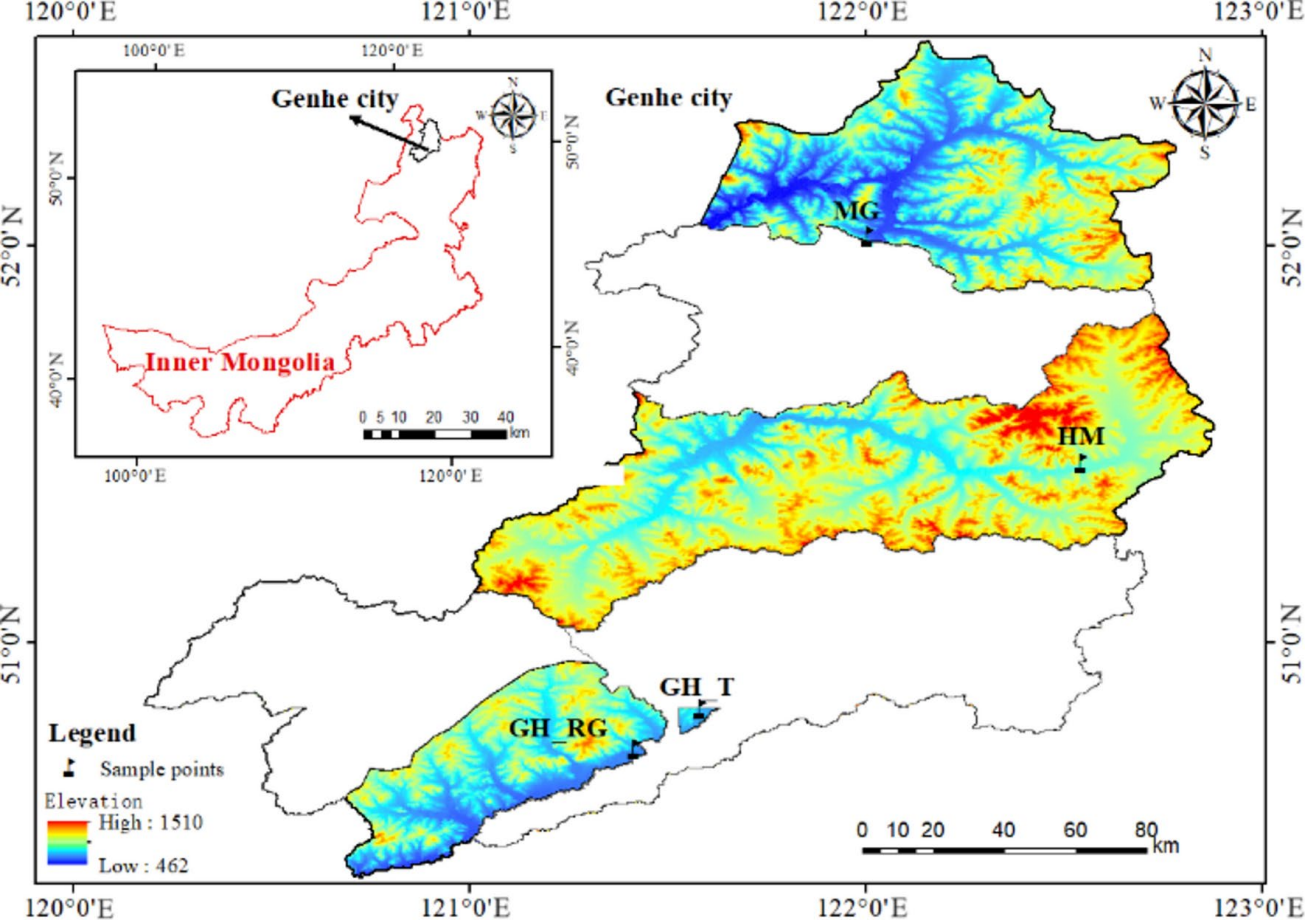
Research area map.

Genhe City (120°12′–122°55′E, 50°20′–52°30′N) administratively oversees three towns (Jinhe Town, Alongshan Town, Mangui Town), one township (Aoluguya Ewenki Ethnic Township), and five sub-district offices (Haolibao, Deerbu, Hedong, Hexi, and Forestry subdistrict offices), with a total area of 20,010 square kilometers. The study area includes Mangui Town (MG, Mangui larch natural forest), Jinhe Town (HM, Hanma larch natural forest), the Forestry subdistrict office (GH_T, Genhe natural larch forest), and Aoluguya Ewenki Ethnic Township (GH_RG, Genhe larch plantation) (Fig. 1)^21^. Genhe City belongs to the cold temperate zone, characterized by a cold and humid climate, poor sunlight conditions, an annual average temperature of − 5.3 °C, and annual average precipitation ranging from 400 to 1562 mm^22^. The altitude ranges from 700 to 1300 m, with an average altitude of 1000 m. The forest area of Genhe City is 1.84 million hectares, accounting for 1.36% of China’s forest area and 11.2% of Inner Mongolia’s forest area, with a forest coverage rate of 91.7%. The main forest vegetation type is Dahurian larch (*Larix gmelinii* (Rupr.) Kuzen), covering 76% of the city’s forest area. This is followed by mixed forests of white birch (*Betula platyphylla* Sukaczev) and Dahurian larch, which account for 19.3%. Other tree species include Scots pine (*Pinus sylvestris* var. mongolica Litv.), aspen (*Populus davidiana* Dode), poplar (Populus L.), and willow (*Salix babylonica* L.).

The study sampling sites are all located within Genhe City, so the climatic characteristics of the four forest land types are generally similar, with some local differences. The average annual temperature in Mangui Town is − 5.8 °C, with an extreme minimum temperature of − 52 °C and an extreme maximum temperature of 31 °C. The average frost-free period is 80 days per year. The forest coverage rate is 92.4%. The climate in Jinhe Town is cold and humid, with long winters and short summers. The frost-free period is 90 days, and the average annual temperature is − 6.1 °C. The historical record shows that the lowest winter temperature has reached − 58 °C. The forest coverage rate is 89.9% (Genhe City People’s Government). The Aoluguya Ewenki Township has a multi-year average temperature of − 6.5 °C, with an extreme maximum temperature of 30.8 °C and an extreme minimum temperature of − 48.8 °C. The annual frost-free period averages between 35 and 85 days.

Genhe City has widespread seasonally discontinuous permafrost, with local areas having permafrost below 30 cm underground^23^. The forest soils in the Genhe River Basin have a simple zonal structure and shallow soil layers, typical of zonal soils—brown coniferous forest soil. The terraces on both sides of the basin feature chernozem and meadow soils^24^.

### Soil sampling and measurement

In April 2023, 76 sampling points were set up within the study area using random sampling. The main soil type of the Hanma natural forest is marsh soil, while the other three sample plots are brown coniferous forest soils. The four sample plots cover a relatively wide range of latitude and longitude, with the average longitude ranging from 121.416558°E to 122.545461°E, and the average latitude ranging from 50.732090°N to 52.023194°N. The average elevations of the four plots are 652.373 m, 850.168 m, 721.542 m, and 703.075 m, respectively. (For detailed information on the basic characteristics of some sampling points, please refer to the Supplementary Material (Tables A1 and A2).

Within each plot, top litter was cleared, and samples were taken from two soil profiles at depths of 0–5 cm and 5–10 cm. The samples were then placed into sealed bags labeled accordingly. The samples were were brought back to the laboratory, where the soil organic carbon content and soil pH value were measured.

A certain amount of treated soil sample was weighed into a ceramic sample boat, and 10% hydrochloric acid was added drop by drop while observing the bubbling. The addition was continued until no bubbles emerged. The acid-treated sample was then placed in a 105 °C oven and dried completely to ensure thorough drying. SOC was measured using the multi N/C series TOC/TN analyzer (multi N/C 3100, Analytik Jena, Germany).

### Data processing

SPSS 26.0 statistical analysis software was used to perform one-way ANOVA, Tukey’s HSD test, and normality test on the organic carbon content in soil samples to analyze the significance of differences in SOC content across different soil layers. The Shapiro–Wilk (S–W) and Kolmogorov–Smirnov (K–S) tests were used to determine whether the SOC content followed a normal distribution.

### Research methodology

#### Coefficient of variation

The coefficient of variation (CV) can measure the overall fluctuation of the data. According to Nielsen’s method, CV is classified into three categories^25^: CV < 10% (weak variation), 10% ≤ CV ≤ 100% (moderate variation), and CV > 100% (strong variation). We use the coefficient of variation to analyze the distribution characteristics of SOC content at sampling points in different forest land types from both horizontal and vertical profile perspectives. The calculation formula is:

1 $${\text{CV}} = \frac{{{\text{SD}}}}{{{\text{Mean}}}} \times 100\%$$

where SD and Mean represent the standard deviation and the mean, respectively.

#### Semivariogram model function

The semi-variogram is a key function in geostatistics for studying soil variability^26^. The semi-variogram is a function of distance h and direction α. A variable distributed in space is called a regionalized variable. The semi-variogram is the mathematical expectation of the squared increments of the regionalized variable ${\text{Z}}({x}_{i})$ and $\text{Z(}{x}_{i}\text{+h)}$, that is, the variance of the increments of the regionalized variable. We use the semi-variogram to examine the spatial heterogeneity of SOC in different soil layers and forest types. The semi-variogram was constructed using GS + 9.0 software, and the data were fitted to a semi-variogram^5^. The best theoretical model was selected based on the coefficient of determination (R^2^) and the residual sum of squares (RSS). Before conducting the geospatial data analysis, the data were standardized using square root transformation. The computation of the semi-variogram is as follows:

2 $${\text{r(h)}} = \frac{1}{{2{\text{N}}({\text{h}})}}\mathop \sum \limits_{i = 1}^{N\left( h \right)} \left[ {Z(x_{i} ) - Z(x_{i} + h)} \right]^{2}$$

where r(h) and N(h) respectively denote the estimated value of the semivariogram at the distance h and the total number of pairs of all points. $Z({x}_{i})$ represents the mean density at the sample point ${x}_{i}$, while $Z({x}_{i}+h)$ denotes the mean density at the sample point ${x}_{i}+h$.

The four important parameters of the semi-variogram include range, nugget (C_0_), sill (C_0_ + C), and nugget effect (C_0_/(C_0_ + C)). The range typically reflects the influence extent of the regionalized variable, the nugget reflects the possible degree of randomness within the regionalized variable, the sill reflects the magnitude of variation in the variable, and the nugget effect describes the degree of spatial variability in the variable. A nugget effect value of less than 25% indicates strong spatial correlation, between 25 and 75% indicates moderate spatial correlation, and greater than 75% indicates weak spatial correlation^27^.

#### Kriging interpolation method

Kriging interpolation, based on the semi-variogram for theoretical and structural analysis, provides an unbiased estimate of regionalized variables within a finite area^28^. Kriging interpolation is one of the main topics in geostatistics and is also referred to as Spatial Best Linear Unbiased Prediction. In this study, the Kriging interpolation tool in ArcGIS software was used to extend the sampling point data to the entire study area. ArcGIS software was utilized to create spatial distribution maps of SOC density and to analyze the vertical distribution characteristics of forest SOC content and the spatial distribution characteristics of SOC density.

## Results

### Distribution characteristics of SOC content at sampling points in different forest types

As shown in Table 1 (0–5 cm), the SOC content in the 0–5 cm soil layer of MG, HM, GH_T, and GH_RG shows significant differences (*P* < 0.05). The order of SOC content is as follows: MG (327.59 g/kg) > HM (301.07 g/kg) > GH_T (133.51 g/kg) > GH_RG (100.98 g/kg), indicating that MG has the highest SOC content. The coefficient of variation in Table 1 shows that MG exhibits weak variation, while HM, GH_T, and GH_RG exhibit moderate variation. The order of the coefficient of variation is as follows: GH_RG (18.07%) > HM (17.68%) > GH_T (13.76%) > MG (5.99%), indicating that the spatial heterogeneity of SOC content in the 0–5 cm layer is greatest under GH_RG. The SOC content of MG (PK-S = 0.2 > 0.05, PS-W = 0.701 > 0.05) follows a normal distribution. The absolute values of skewness and kurtosis for the SOC content in HM, GH_T, and GH_RG are less than 10 for skewness and less than 3 for kurtosis, respectively, indicating that the SOC content in these three forest types essentially follows a normal distribution^29^.

**Table 1:** Descriptive statistics of SOC content in 0-5 cm soil layer in the study area.

Statistical measures	MG			HM			GH_T			GH_RG	
	0–5 cm	5–10 cm		0–5 cm	5–10 cm		0–5 cm	5–10 cm		0–5 cm	5–10 cm
Minimum value (g/kg)	285.10	313.10		211.30	50.76		79.87	50.87		54.36	31.78
Maximum value (g/kg)	377.30	332.40		362.69	312.90		160.93	89.76		164.30	61.78
Mean (g/kg)	327.59a	322.71a		301.07b	216.10b		133.51c	75.48c		100.98d	44.22d
Standard deviation	19.62	3.64		53.23	56.77		18.37	7.92		18.25	8.25
Variance	384.95	13.27		2832.96	3223.23		337.28	62.72		333.09	68.02
CV (%)	5.99	1.13		17.68	26.27		13.76	10.49		18.07	18.66
Skewness	0.397	− 0.471		− 0.201	− 0.001		− 1.084	− 1.009		1.049	0.746
Kurtosis	0.237	0.845		− 1.650	− 0.058		0.993	1.439		2.705	− 0.597
P_K–S_	0.200	0.200		0.000	0.035		0.007	0.200		0.000	0.005
P_S–W_	0.701	0.188		0.000	0.009		0.002	0.002		0.000	0.000

Different lowercase letters in the table indicate significant differences in SOC content among different types of forests (*P* < 0.05). MG represents the Mangui Natural Forest; HM represents the Hanma Natural Forest; GH_T represents the Genhe Natural Forest; GH_RG represents the Genhe Plantation Forest.

As shown in Table 1 (5–10 cm), the SOC content in the 5–10 cm soil layer of MG, HM, GH_T, and GH_RG has significant differences (*P* < 0.05). The range of SOC content in the four forest land types is from 44.22 to 322.71 g/kg. The order of SOC content is as follows: MG (322.71 g/kg) > HM (216.10 g/kg) > GH_T (75.48 g/kg) > GH_RG (44.22 g/kg). Among them, the SOC content of MG is 49.33%, 327.54%, and 629.78% higher than that of the HM, GH_T, and GH_RG soils, respectively. The coefficient of variation in Table 1 shows that MG exhibits weak variation, while HM, GH_T, and GH_RG exhibit moderate variation. The order of the coefficient of variation is as follows: HM (26.27%) > GH_RG (18.66%) > GH_T (10.49%) > MG (1.13%), indicating that the spatial heterogeneity of SOC content in the 5–10 cm layer is strongest under HM. Based on skewness, kurtosis, and PK-S, PS-W, the SOC content in the four forest types essentially follows a normal distribution.

**Fig. 2:**
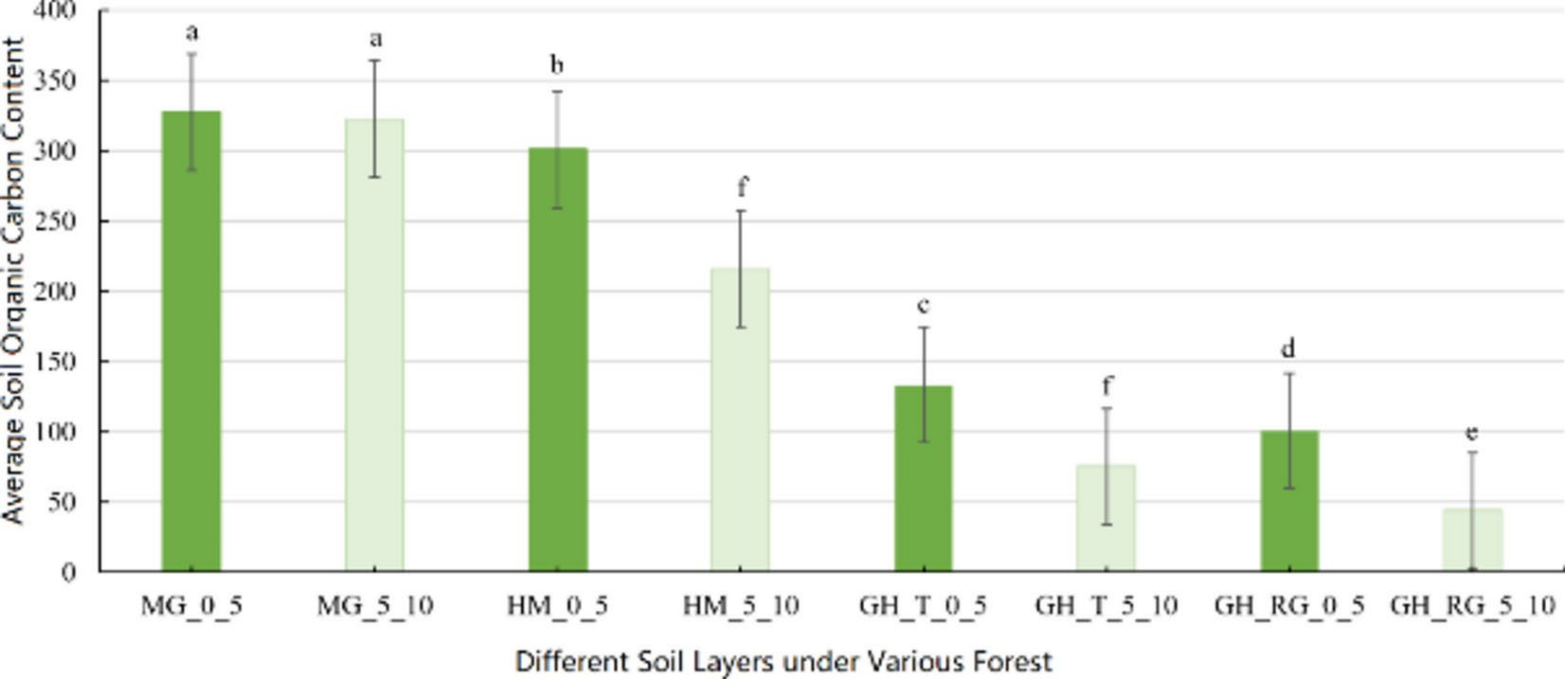
Average SOC in different soil layers under different forest types. *Note*: Different lowercase letters in the table indicate significant differences in SOC content among different types of forests (*P* < 0.05). MG_0_5 represents Mangui 0–5 cm sampling point, MG_5_10 represents Mangui 5–10 cm sampling point; HM_0_5 represents Hanma 0–5 cm sampling point, HM_5_10 represents Hanma 5–10 cm sampling point; GH_T_0_5 represents Genhe Natural Forest 0–5 cm sampling point, GH_T_5_10 represents Genhe Natural Forest 5–10 cm sampling point; GH_RG_0_5 represents Genhe Plantation Forest 0–5 cm sampling point, GH_RG_5_10 represents Genhe Plantation Forest 5–10 cm sampling point.

Figure 2 indicates that the vertical distribution characteristics of SOC content are apparent among the three forest types, HM, GH_T, and GH_RG. There are significant differences in SOC content between the 0–5 and 5–10 cm soil layers in HM, GH_T, and GH_RG, while MG does not show significant differences between these two layers.

**Fig. 3:**
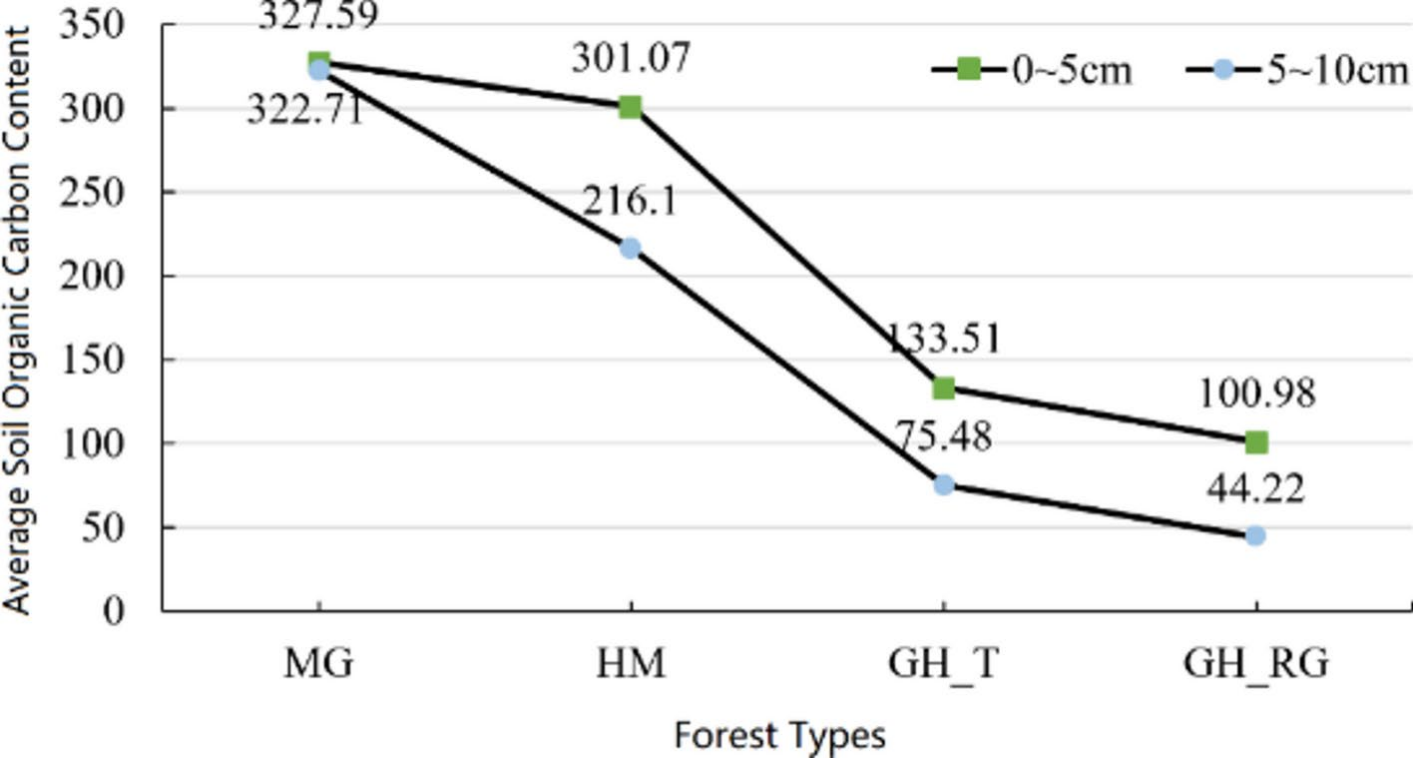
Average value of SOC content at different depths. *Note*: MG represents Mangui; HM represents Hanma; GH_T represents Genhe Natural Forest; GH_RG represents Genhe Plantation Forest.

Figure 3 shows that the SOC content of the four forest types decreases with increasing soil depth. The SOC content in MG shows little change of 4.88 g/kg; the rates of decline in top SOC content for GH_T and GH_RG are similar, at 58.03 g/kg and 56.76 g/kg, respectively; HM has the fastest decline rate at 84.97 g/kg.

### Analysis of spatial heterogeneity of SOC content in top soil

As mentioned in Section 3.1, the observed values of the sample points follow a normal or near-normal distribution, and these data were used to construct the semi-variogram model^5^.

As shown in Table 2 and Fig. A1-a (Fig. A1-a in the Supplementary Material), the 0–5 cm soil layer (R^2^ = 97.3%, RSS = 0.110) shows a high degree of fit, indicating that the theoretical model can accurately reflect the spatial structure characteristics of SOC content. As shown in Table 2 and Fig. A1-b (Fig. A1-b in the Supplementary Material), the 5–10 cm soil layer (R^2^ = 82.0%, RSS = 0.976) also shows a high degree of fit, indicating that the theoretical model can accurately reflect the spatial structure characteristics of SOC content. The best-fit models for the semi-variograms of SOC content in the 0–5 cm and 5–10 cm layers are Gaussian models. The nugget values for the 0–5 cm and 5–10 cm layers are 0.004 and 0.001, respectively. The nugget effect values for both layers are above 75%, indicating very weak spatial autocorrelation. The range values for the semi-variogram models for the two soil layers are 39.283 m and 18.291 m, respectively, suggesting that the SOC content in different soil layers within the study area exhibits spatial autocorrelation within these ranges, reflecting the maximum spatial autocorrelation distance in the data. Beyond this range, spatial autocorrelation ceasess^27^.

**Table 2:** Isotropic semi-variance function model and its parameters of SOC content in different soil layers.

Soil depth (cm)	Model theory	C_0_	C_0_ + C	[C_0_/(C_0_ + C)] (%)	Range (m)	R^2^	RSS
0–5	Gaussian	0.004	2.01	99.80	39.28	97.30	0.11
5–10	Gaussian	0.001	1.53	99.90	18.29	82.00	0.98

As shown in Table 3 and Fig. A2 (Fig. A2 in the Supplementary Material), the best-fit models for the semi-variograms of SOC content in the 0–5 cm and 5–10 cm layers for MG, HM, GH_T, and GH_RG are all Gaussian models. The R^2^ values for different soil layers in MG are all above 0.95, and the RSS values are very small, indicating a high degree of fit, showing that the two models can well reflect the spatial structure characteristics of SOC content. HM has a high degree of fit in different soil layers, with high R^2^ values (95.9%, 92.0%), but relatively high residual values (2.81 and 7.43), indicating that the two models can well reflect the spatial structure characteristics of SOC content. For GH_T in different soil layers, the R^2^ values are 82.9% and 78.3%, respectively, but the RSS values are very small, indicating an average degree of fit, showing that the two models have an average ability to reflect the spatial structure of SOC content. GH_RG has an average degree of fit in the 0–5 cm soil layer, with an R^2^ of 87.8% and a small RSS of 0.219, indicating an average ability to reflect the spatial structure characteristics of SOC content. GH_RG has a good degree of fit in the 5–10 cm soil layer, with a high R^2^ and small RSS, indicating a good ability to reflect the spatial structure characteristics of SOC content.

**Table 3:** Isotropic semi-variance function model and its parameters of SOC content in different forest types.

Forest types	Soil depth (cm)	Model theory	C_0_	C_0_ + C	[C_0_/(C_0_ + C)] (%)	Range (m)	R^2^	RSS
MG	0–5	Gaussian	0.0030	0.44	99.30	33.01	97.10	7.654E−03
	5–10	Gaussian	0.00001	0.01	99.90	20.32	95.70	9.615E−06
HM	0–5	Gaussian	0.0100	5.03	99.80	36.37	95.90	2.81
	5–10	Gaussian	0.0100	6.88	99.90	26.36	92.00	7.43
GH_T	0–5	Gaussian	0.0010	0.56	99.80	36.01	82.90	0.09
	5–10	Gaussian	0.0001	0.14	99.90	26.66	78.30	0.01
GH_RG	0–5	Gaussian	0.0740	1.14	93.50	31.86	87.80	0.22
	5–10	Gaussian	0.0210	0.70	97.00	50.87	96.30	9.547E−03

MG represents Mangui; HM represents Hanma; GH_T represents Genhe Natural Forest; GH_RG represents Genhe Plantation Forest; same below.

The nugget values for different forest types in the 0–5 cm soil layer range from 0.001 to 0.074, whereas in the 5–10 cm soil layer, they range from 0.00001 to 0.021, indicating that the 0–5 cm soil layer has greater internal randomness and more significant differences in SOC content between different forest types. In comparison, the 5–10 cm soil layer has lower internal randomness in SOC content. The nugget effect values for SOC content in different forest types and soil layers are all above 75%, indicating very weak spatial autocorrelation. The range values for the semi-variogram models of SOC content across forest types and soil layers range from 20.317 to 50.87 m, suggesting that the SOC content in different forest types exhibits spatial autocorrelation within these range values. The smallest range value is observed in in the 5–10 cm soil layer of MG, indicating a more fragmented spatial distribution of SOC content^30^.

### Analysis of spatial distribution patterns of SOC content in top soil

**Fig. 4:**
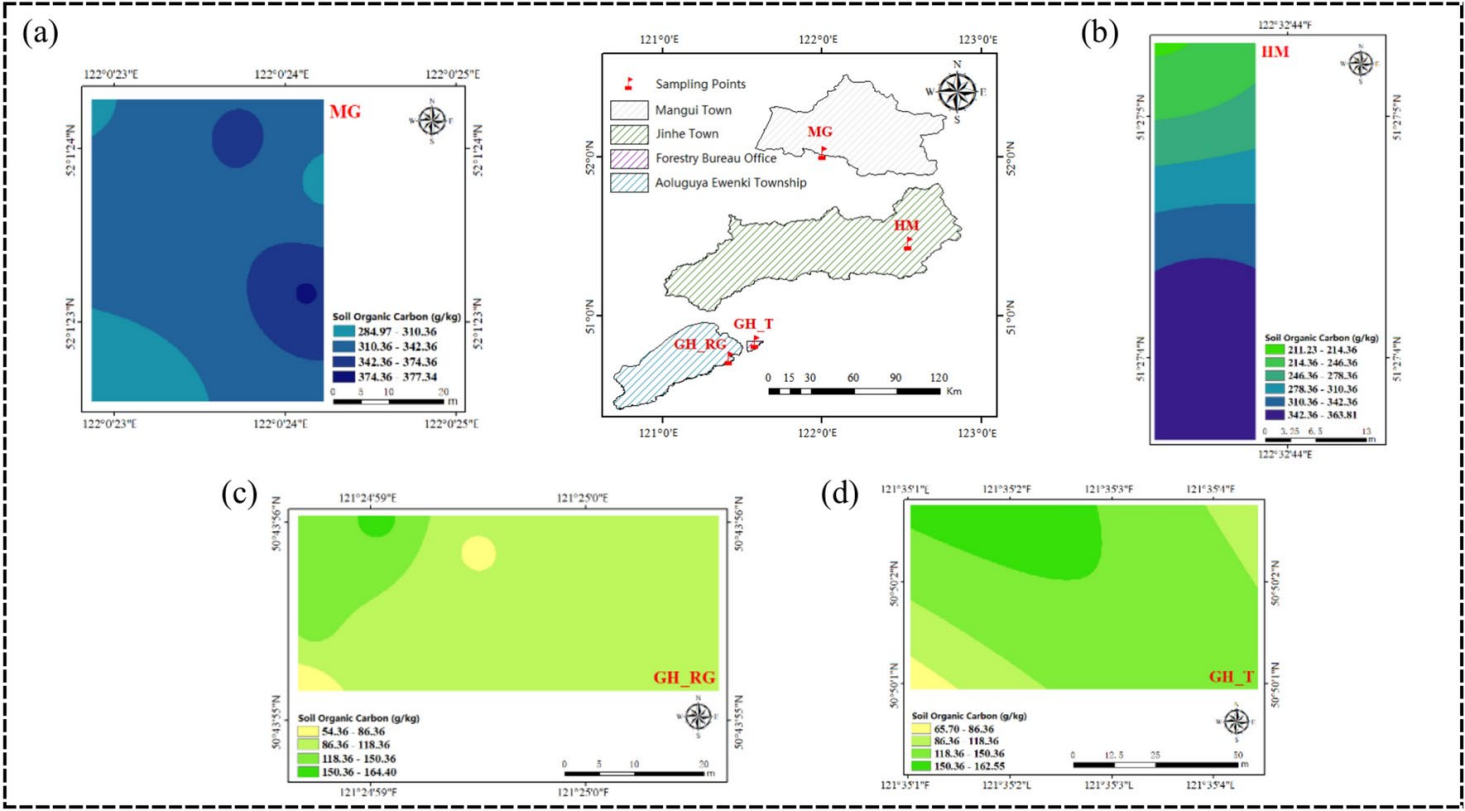
SOC Content in the Horizontal Layer Study Area (0–5 cm). *Note*: **a** represents Mangui; **b** represents Hanma; **c** represents Genhe Plantation Forest; **d** represents Genhe Natural Forest. The colors represent the relative levels of SOC content in each plot: yellow indicates low SOC content, green indicates medium SOC content, and blue indicates high SOC content. The variation in color intensity further reflects the level of organic carbon content, with darker colors indicating higher organic carbon content in each plot.

**Fig. 5:**
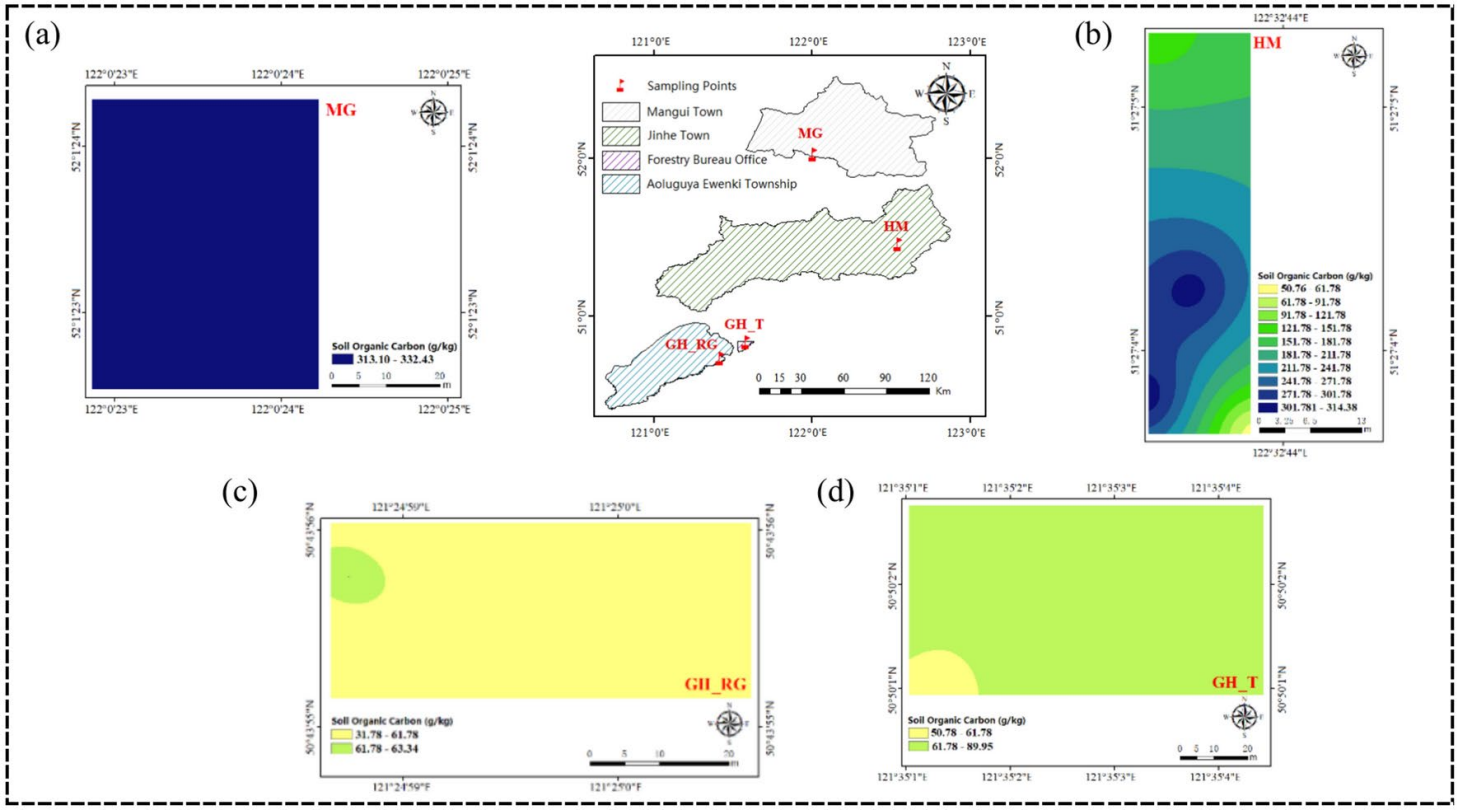
SOC Content in the Horizontal Layer Study Area (5–10 cm). *Note*: **a** represents Mangui; **b** represents Hanma; **c** represents Genhe Plantation Forest; **d** represents Genhe Natural Forest. The colors represent the relative levels of SOC content in each plot: yellow indicates low SOC content, green indicates medium SOC content, and blue indicates high SOC content. The variation in color intensity further reflects the level of organic carbon content, with darker colors indicating higher organic carbon content in each plot.

Figures 4 and 5 shows that the distribution of SOC content in the 0–5 cm and 5–10 cm soil layers across the study area is generally similar, both exhibiting an increasing trend with latitude. As shown in Figs. 4a and 5a, the soil organic carbon content in the 0–5 cm soil layer of the MG sampling area predominantly ranges from 310.36 to 342.36 g/kg, with smaller portions ranging from 284.97 to 310.36 g/kg, 342.36 to 374.37 g/kg, and 374.36 to 377.34 g/kg. In the 5–10 cm soil layer, the soil organic carbon content in the MG sampling area primarily ranges from 313.10 to 332.43 g/kg. However, as shown in Figs. 4b and 5b, in the HM sampling area, the SOC content in the 0–5 cm soil layer increases as latitude decreases, while in the 5–10 cm soil layer, the SOC content distribution shows a high center with decreasing values toward the edges, with some regions displaying a low center with increasing values toward the edges. This variation may be related to factors such as vegetation and topography. The average pH values of the soils in MG, GH_T, and GH_RG are above 5, while the average pH value in HM is below 5. Moreover, the average altitude of HM is higher than that of MG, GH_T, and GH_RG, indicating that altitude has an impact on top SOC content. As shown in Figs. 4c and 5c, the soil organic carbon content in the 0–5 cm soil layer of the GH_RG sampling area predominantly ranges from 86.36 to 118.36 g/kg. In the 5–10 cm soil layer, the soil organic carbon content in the GH_RG sampling area primarily ranges from 31.78 to 61.78 g/kg. As shown in Figs. 4d and 5d, the soil organic carbon content in the 0–5 cm soil layer of the GH_T sampling area predominantly ranges from 118.36 to 150.36 g/kg. In the 5–10 cm soil layer, the soil organic carbon content in the GH_T sampling area primarily ranges from 61.78 to 89.95 g/kg. From the perspective of natural versus artificial forests, the overall SOC content in GH_T is higher than that in GH_RG.

**Fig. 6:**
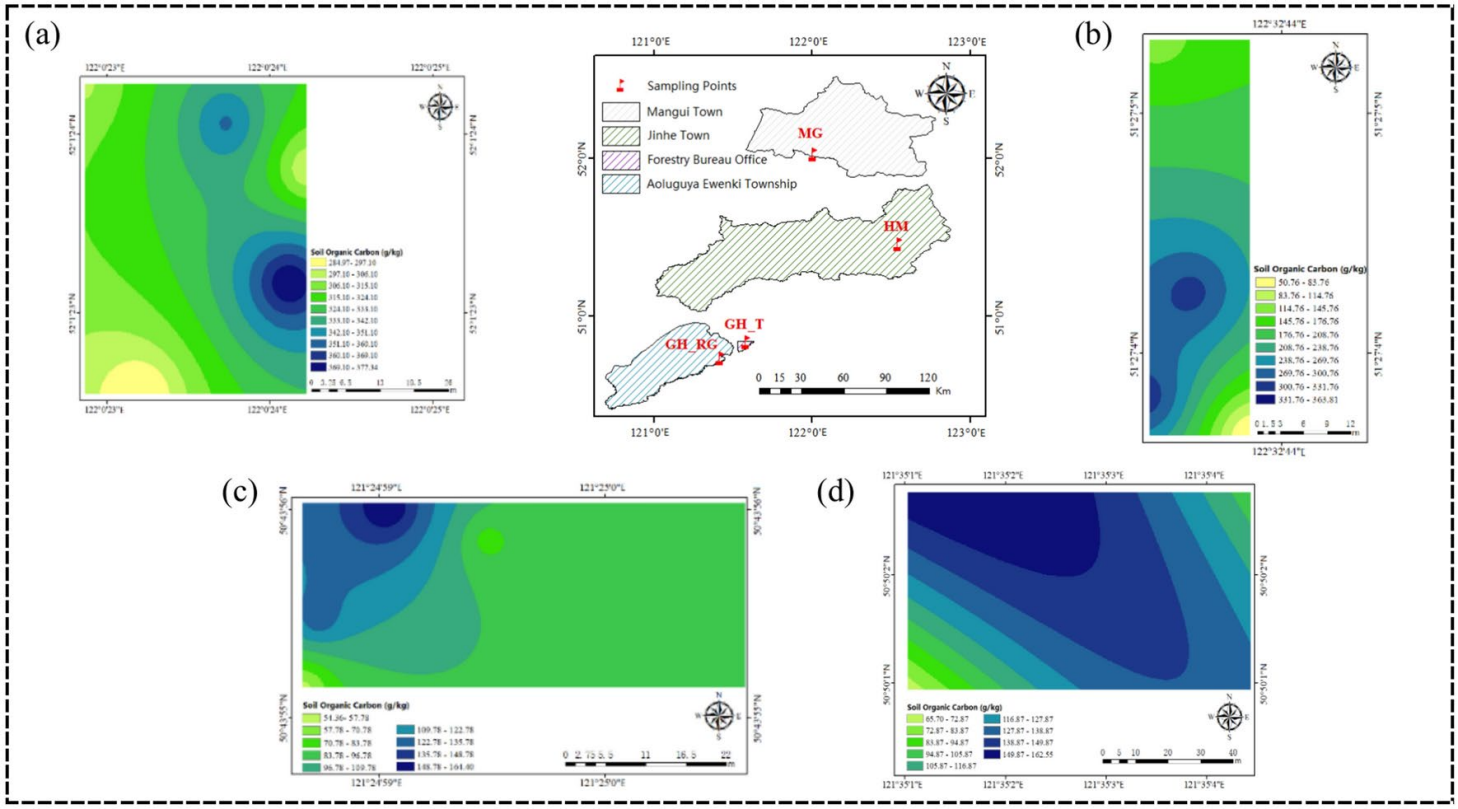
SOC Content at Different Soil Depths in the Study Area. (0–5 cm). *Note*: **a** represents Mangui; **b** represents Hanma; **c** represents Genhe Plantation Forest; **d** represents Genhe Natural Forest. The colors represent the relative levels of SOC content in each plot: yellow indicates low SOC content, green indicates medium SOC content, and blue indicates high SOC content. The variation in color intensity further reflects the level of organic carbon content, with darker colors indicating higher organic carbon content in each plot.

**Fig. 7:**
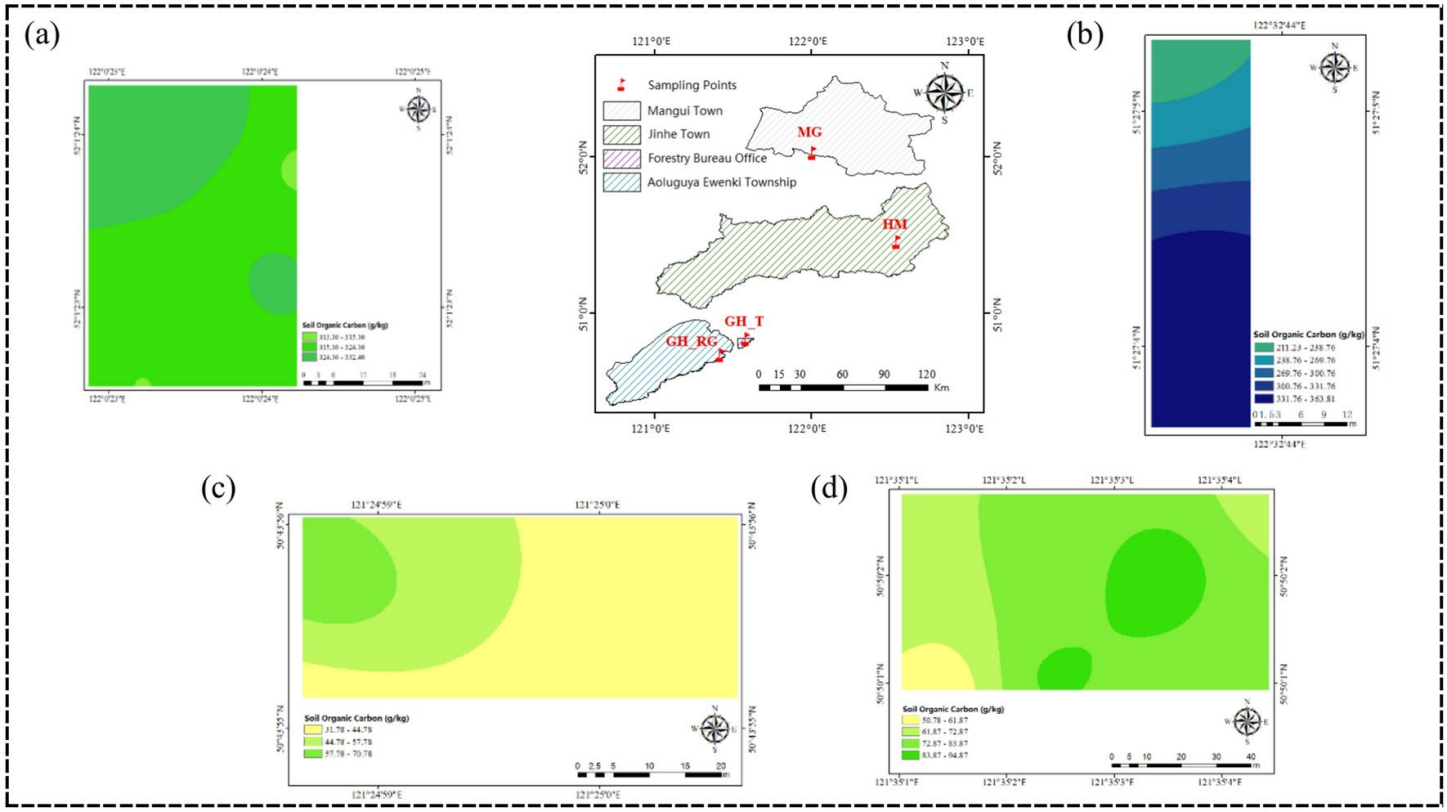
SOC Content at Different Soil Depths in the Study Area. (5–10 cm). *Note*: **a** represents Mangui; **b** represents Hanma; **c** represents Genhe Plantation Forest; **d** represents Genhe Natural Forest. The colors represent the relative levels of SOC content in each plot: yellow indicates low SOC content, green indicates medium SOC content, and blue indicates high SOC content. The variation in color intensity further reflects the level of organic carbon content, with darker colors indicating higher organic carbon content in each plot.

Figures 6a and 7a show that the SOC content in the 0–5 cm soil layer of MG_5 has a bimodal contour distribution, while in the 5–10 cm soil layer, MG_10 has a more concentrated and uniform distribution. Figure 6b and 7b show that HM_5 has a stratified distribution of SOC content, while HM_10 shows bimodal and basin-like contour distributions. As shown in Figs. 6c and 7c, GH_RG_5 has a more concentrated distribution in the east with a more uniform distribution in the west, while GH_RG_10 shows a ring-like distribution with decreasing values from the center outward. Figures 6d and 7d show that GH_T_5 has a ridge-like contour distribution, whereas GH_T_10 shows a concentrated distribution with central high and decreasing values toward the edges. Research indicates that SOC content generally decreases with increasing soil depth.

## Discussion

### Distribution of top SOC content and environmental factors in different forest types

Wang et al.^31^ find that forest SOC exhibits a clear latitudinal pattern. They also point out that the primary factor influencing this latitudinal distribution of SOC components is climate, with soil texture being a secondary factor. In the study of the distribution characteristics of SOC content at sampling points across different forest types, the ordering of SOC content at depths of 0–5 cm and 5–10 cm is the same, namely MG > HM > GH_T > GH_RG. Among them, MG and GH_T have the same tree species and soil type, yet there is a significant difference in their SOC content, which may be related to the difference in latitude. Du et al.^32^ find that the low temperature conditions in permafrost regions effectively suppress the decomposition of organic matter, thereby promoting the accumulation of organic carbon. Thus, the higher SOC content in the MG region may be related to its higher latitude and colder environment, which slows down soil microbial activity and the rate of organic carbon decomposition. The research of Li^33^ shows that the anaerobic environment in permafrost soils plays a critical role in maintaining the long-term stability of organic carbon. Although this study does not directly address anaerobic environments, the combined effects of latitude and low temperature in the MG region may contribute to the higher SOC content. This trend aligns with our finding that surface soil SOC generally increases with latitude.

In terms of the effect of altitude on SOC, our results are consistent with Wu et al.^13^, showing that the SOC mass fraction in forest regions generally increases with elevation. Chiluveri et al.^34^ measured the soil’s physical and chemical properties in different forest areas at various altitudes in the Garhwal Himalayas, indicating that higher altitudes correspond to higher SOC and nitrogen reserves in the top soil. However, the HM sampling area shows different results, which may be related to factors such as vegetation, topography, or altitude. Similarly, Xia et al.^35^ observe a decreasing trend in SOC content with increasing altitude and find that SOC content can reach its maximum at certain slope ranges, with the highest values being found on shady slopes. The SOC variation trend in the HM area differs from that observed at other study sites, possibly due to factors such as tree species, elevation, and slope. Moreover, SOC content in HM decreases most rapidly with increasing soil depth, possibly due to the influences of soil type.

Using the coefficient of variation, the study on the spatial heterogeneity of top SOC content finds that the 0–5 cm and 5–10 cm soil layers have the weakest spatial heterogeneity of SOC content in MG, possibly due to the sampling from areas without permafrost. In the 0–5 cm soil layer, GH_RG exhibits the strongest spatial heterogeneity in SOC content, likely due to human intervention. In the 5–10 cm soil layer, HM shows the strongest spatial heterogeneity in SOC content, which may be related to soil type.

### Spatial distribution pattern of top SOC content

The SOC content at the four sampling points ranges from 54.36 to 377.34 g/kg, which is similar to the findings of Zhang et al.^36^, where the SOC range is between 2.57 and 396.50 g/kg. Both studies are conducted in areas with seasonally discontinuous permafrost. Aboveground vegetation and underground root systems are the primary sources of SOC in permafrost forest soils^37^. These plants and their roots contain substantial organic matter, contributing to top SOC content. The MG sampling area’s soil layer is relatively thin and contains permafrost, which may result in top SOC levels significantly higher than those in the GH_T and GH_RG sampling areas. The HM sampling area consists of marsh soil with a peat top layer, rich in organic matter, potentially leading to top SOC levels, again significantly higher than in the GH_T and GH_RG areas. The composition of SOC includes fine roots with a diameter of ≤ 2 mm, which are difficult to distinguish from underground biomass. Combined with the abundance of litter in the sampling areas, this results in top SOC content. The top SOC content in the 0–5 cm soil layer ranges from 374.36 to 377.34 g/kg, while in the 5–10 cm soil layer, it ranges from 313.1 to 332.43 g/kg.

Our study demonstrates that organic carbon content in the top soil (0–10 cm) generally decreases with increasing soil depth, consistent with the findings of Wang et al.^38^ and Qin et al.^39^. Thesurface layer of forest soil contains litter, has a loose soil structure, good aeration, and intense biological activity. The decomposition of litter by microorganisms forms a large amount of humus, leading to higher nutrient levels in the upper soil layer^40^. Meng et al.^41^ find that the SOC in all community types in Beijing urban forests decreases with increasing soil depth, with the 0–20 cm soil layer having the greatest impact on the diversity of understory plants. However, in some localized areas of the MG sampling site, the top SOC content increases, likely due to vegetation types. Zhang et al.^42^ also find that top SOC content decreased with increasing soil depth, though with some local increases. Additionally, Ma et al.^43^ discover that in different habitats, SOC content first increases then decreases, and then increases again with soil depth.

## Conclusion

The results indicate that in the four forest types studied, the organic carbon content in the top soil decreases with increasing soil depth. Through semi-variogram analysis, the results of this study revealed the spatial autocorrelation characteristics of SOC content. The results show that SOC content exhibits high spatial clustering within certain spatial scales, which is important for predicting and managing soil carbon resources. Horizontally, SOC content in the 0–5 cm and 5–10 cm soil layers increases with latitude, likely influenced by changes in environmental factors such as temperature and humidity. In some regions, the 0–5 cm soil layer in HM shows increasing SOC content with decreasing latitude, while the 5–10 cm layer shows a high center with lower edges, and in other regions, the pattern is reversed. Vertically, SOC content generally decreases with increasing soil depth in the 0–5 cm layer, though some areas show an increase. The 5–10 cm layer also exhibits a decreasing trend.

## Supplementary Information

Supplementary Information.

## Data Availability

The datasets generated and/or analyzed during the current study are not publicly available due to [the data being used for further research by the research team and not publicly disclosed at this time], but are available from the corresponding author on reasonable request.
