# Supplementary material for: Distribution characteristics of top SOC in different forest types of Genhe in the Greater Khingan Range of Inner Mongolia
Source: Sci Rep. 2025 Feb 25;15:6736. doi: 10.1038/s41598-025-88170-6 (PMC11862182; doi:10.1038/s41598-025-88170-6)
Supplement: Supplementary file 1 — Supplementary Information. [file 41598_2025_88170_MOESM1_ESM.docx]

**Supplementary Material**

Table A1 Basic characteristics of sampling points

| Overview of Various Indicators | Mangui Natural Forest | Hanma Natural Forest | Genhe Natural Forest | Genhe Plantation Forest |
| --- | --- | --- | --- | --- |
| Main Soil Types | Brown Coniferous Forest Soil | Bog Soil | Brown Coniferous Forest Soil | Brown Coniferous Forest Soil |
| Average Longitude (°) | 122.006550 | 122.545461 | 121.584102 | 121.416558 |
| Average Latitude (°) | 52.023194 | 51.451272 | 50.833870 | 50.732090 |
| Average Elevation(m) | 652.373 | 850.168 | 721.542 | 703.075 |
| Average SOC5(g/kg) | 327.59 | 301.07 | 133.51 | 100.98 |
| Average SOC10(g/kg) | 322.71 | 216.10 | 75.48 | 44.22 |

Note: SOC5 refers to the soil organic carbon content at a depth of 0-5 cm, and SOC10 refers to the soil organic carbon content at a depth of 5-10 cm; the average values of SOC5 and SOC10 are obtained from laboratory tests.

Table A2 Basic information about some sampling points

| Sampling point | Longitude (°) | Latitude (°) | Elevation(m) |
| --- | --- | --- | --- |
| Genhe Natural Forest_1 | 121.58456409° | 50.83409824° | 725.031 |
| Genhe Natural Forest_2 | 121.58430384° | 50.83391002° | 731.109 |
| Genhe Natural Forest_3 | 121.58398164° | 50.83361482° | 727.948 |
| Genhe Natural Forest_4 | 121.58361960° | 50.83369080° | 734.873 |
| Genhe Plantation Forest_1 | 121.41683825° | 50.73220738° | 712.458 |
| Genhe Plantation Forest_2 | 121.41652022° | 50.73216298° | 717.167 |
| Genhe Plantation Forest_3 | 121.41629505° | 50.73198218° | 710.900 |
| Genhe Plantation Forest_4 | 121.41639951° | 50.73220313° | 713.767 |
| Hanma Natural Forest_1 | 122.54540229° | 51.45105471° | 862.200 |
| Hanma Natural Forest_2 | 122.54551831° | 51.45101611° | 861.464 |
| Hanma Natural Forest_3 | 122.54545363° | 51.45118157° | 859.662 |
| Hanma Natural Forest_4 | 122.54540997° | 51.45147363° | 863.763 |
| Mangui Natural Forest_1 | 122.00635215° | 52.02341012° | 663.883 |
| Mangui Natural Forest_2 | 122.00659425° | 52.02335053° | 661.720 |
| Mangui Natural Forest_3 | 122.00672928° | 52.02327589° | 665.267 |
| Mangui Natural Forest_4 | 122.00645020° | 52.02292964° | 665.396 |

a) b)

Fig. A1. Isotropic semi-variance function model of soil organic carbon content in different soil layers.

Note: a) 0-5 cm Soil Layer; b) 5-10 cm Soil Layer.

a) b) c)

d) e) f)

g) h)

Fig. A2. soil organic carbon content isotropic semi-variance function model.

Note: a) MG_0-5 cm Soil Layer; b) MG_5-10 cm Soil Layer; c) HM_0-5 cm Soil Layer; d) HM_5-10 cm Soil Layer; e) GH_T_0-5 cm Soil Layer; f) GH_T_5-10 cm Soil Layer; g) GH_RG_0-5 cm Soil Layer; h) GH_RG_5-10 cm Soil Layer.
